# Analysis of Selected Salivary Adipokines and Cytokines in Patients with Obesity—A Pilot Study

**DOI:** 10.3390/ijms24044145

**Published:** 2023-02-18

**Authors:** Lucyna Ostrowska, Joanna Smarkusz-Zarzecka, Agnieszka Gornowicz, Karolina Lendzion, Beata Zyśk, Damian Pogodziński

**Affiliations:** 1 Department of Dietetics and Clinical Nutrition, Medical University of Bialystok, 15-054 Bialystok, Poland; 2 Department of Biotechnology, Medical University of Bialystok, 15-089 Bialystok, Poland

**Keywords:** obesity, saliva, body composition, proinflammatory cytokines, proinflammatory adipokines, IL-6, IL-1β, resistin, MMP-9, MMP-2

## Abstract

Obesity is a chronic, progressive and relapsing disease that produces many adverse health, social and economic effects. The aim of the study was to analyse the concentrations of selected proinflammatory parameters in the saliva of obese and normal body weight individuals. The study included 116 people divided into two groups: the study group (*n* = 75, subjects with obesity) and the control group (*n* = 41, individuals with normal body weight). Bioelectrical impedance analysis was performed, and saliva samples were collected from all study participants to determine the concentrations of selected proinflammatory adipokines and cytokines. Statistically significantly higher concentrations of MMP-2, MMP-9 and IL-1β were found in the saliva of obese women compared to women with normal body weight. Furthermore, statistically significantly higher concentrations of MMP-9, IL-6 and resistin were observed in the saliva of obese men compared to men with normal body weight. Higher concentrations of selected proinflammatory cytokines and adipokines were found in the saliva of obese individuals compared to individuals with normal body weight. It is likely that higher concentrations of MMP-2, MMP-9 and IL-1β can be detected in the saliva of obese women compared to non-obese women, while higher concentrations of MMP-9, IL-6 and resistin can be found in the saliva of obese men compared to non-obese men, which suggests that further research to confirm our observations and determine the mechanisms of development of metabolic complications associated with obesity depending on gender is needed.

## 1. Introduction

Obesity is a chronic, progressive, relapsing disease that produces many adverse health, psychological, social and economic effects. In 2013, the Member States of the World Health Assembly agreed to a set targets which include halting the rise in obesity at 2010 levels by 2025 [1]. However, the World Obesity Federation Annual Report 2020 indicates that the probability of most countries achieving this target is less than 10%. Furthermore, it is forecast that the prevalence of obesity will increase from 11.4% to 17.5% in the years 2010–2030, with over 1 billion people living with obesity globally by 2030. One in five women and one in seven men will be obese [2]. According to the Organization for Economic Cooperation and Development (OECD) data from 2019, obesity and its complications will contribute to approximately 92 million premature deaths in OECD, Group 20 (G20) and European Union (EU28) member countries in the next 30 years. Furthermore, life expectancy will decrease by 0.9–4.2 years in the above countries within the same period of time [3]. Therefore, routine monitoring of obese individuals aimed at early identification of those at risk for developing metabolic complications should be introduced. 

It is crucial that the mechanisms underlying metabolic disturbances in the obese in relation to inflammatory processes involving white adipose tissue are fully understood. Adipose tissue cells secrete adipokines and cytokines that induce immune cell infiltration, thus promoting the proinflammatory phenotype. Furthermore, they produce not only paracrine and autocrine but also endocrine effects, and consequently affect both inflammation in adipose tissue and systemic inflammation. They can cause metabolic diseases, including hypertension, atherogenic dyslipidemia, insulin resistance, non-alcoholic fatty liver disease, type 2 diabetes, and may be an indirect cause of many chronic diseases, i.e., cancer or cardiovascular diseases [4].

The use of saliva for early detection of inflammation in the oral cavity and diagnosis of periodontal diseases is well established [5]. However, changes in the concentration of many salivary parameters may also be useful as biomarkers for other diseases, including caries [6]. Systematic reviews and meta-analyses conducted in recent years indicate that saliva can be used for the diagnosis and surveillance of a number of systemic diseases, including inflammatory bowel diseases [7], oral cancer and systemic oncological diseases [8]. Saliva could be a valuable diagnostic tool in everyday clinical practice and in screening large populations due to the non-invasiveness of the test and a lower risk of infection compared to serum [5].

In one of our previous studies, we investigated proinflammatory adipokines and cytokines which may play a role in metabolic disturbances in the obese. A review of the available literature suggests that research on resistin and interleukin 6 (IL-6) should be continued since the results of previous studies are inconclusive [9]. IL-6 is a multidirectional cytokine, the activity of which depends on the source of its expression. When secreted by adipose tissue cells in response to the development of obesity, it causes increased accumulation of macrophages in the tissue [10]. On the other hand, elevated resistin concentration is involved in the pathogenesis of inflammation and the development of insulin resistance, type 2 diabetes, hypertension, atherogenic dyslipidemia and atherosclerosis [11].

Matrix metalloproteinase-2 (MMP-2) and MMP-9 as well as interleukin 1-beta (IL-1β) may be potential salivary biomarkers for risk prediction of metabolic disturbances in the obese. To date, few studies have been conducted on the subject. However, they should be continued as promising results have been obtained [9]. Matrix metalloproteinases (MMPs), and more specifically MMP-2 and MMP-9 gelatinases, have been implicated in the development of atherosclerosis [12]. IL-1β contributes to the development of insulin resistance that accompanies obesity [13]. 

Many previous studies that investigated obesity markers were based mainly on body mass index (BMI) as a diagnostic criterion for this disease. Future research should also assess the content and distribution of adipose tissue which correlates, to a greater extent, with the risk of metabolic disturbances [4,9].

The aim of the study was to analyse the concentrations of selected proinflammatory parameters in the saliva of obese and normal body weight individuals. 

## 2. Results

The characteristics of selected anthropometric and body composition parameters of women (*n* = 77) from the study group (*n* = 46) and women from the control group (*n* = 31), and men (*n* = 39) from the study group (*n* = 29) and men from the control group (*n* = 10) are presented in Table 1.

Comparison of women from the study and control groups revealed statistically significant differences for age, body weight, BMI (body mass index), waist circumference, hip circumference, WHR (waist-to-hip ratio), total body fat (kg and %), VAT (visceral adipose tissue, cm^2^ and %), SAT (subcutaneous adipose tissue, cm^2^ and %) and the VAT/SAT ratio. Similar results were obtained for men, but no statistically significant differences were found for body height, percentage of visceral and subcutaneous fat and the VAT/SAT ratio.

Next, concentrations of selected proinflammatory cytokines and adipokines in the saliva of participants from the study and control groups were assessed and the results are presented in Table 2. 

Statistically significantly higher concentrations of MMP-2, MMP-9 and IL-1β were found in the saliva of obese women compared to women with normal body weight. Furthermore, statistically significantly higher concentrations of MMP-9, IL-6 and resistin were observed in the saliva of obese men compared to men with normal body weight.

Next, correlations between selected anthropometric/body composition parameters and cytokines/adipokines in the saliva of women from the study and control groups (*n* = 77) were analysed, and the results are presented in Table 3 and Supplementary Tables S1 and S2.

Statistically significant positive correlations were found between BMI and the salivary concentrations of MMP-2, MMP-9, resistin and IL-1β. A similar relationship was also observed for hip circumference and total body fat (kg). Statistically significant positive correlations were also found between waist circumference and serum concentrations of MMP-2, MMP-9 and IL-1β. A similar relationship was also observed for the percentage of total body fat and subcutaneous fat (SAT (cm^2^)). No statistically significant correlations were found between the WHR index and the salivary concentrations of cytokines/adipokines. Statistically significant positive correlations were found between VAT (cm^2^) and the salivary concentrations of MMP-2, resistin and IL-1β, whereas the percentage of VAT showed a statistically significant positive correlation only with the concentration of MMP-2 in saliva. A similar relationship was observed for the VAT/SAT ratio. A statistically significant negative correlation was found only between SAT (%) and the concentration of MMP-9 in saliva. There were no statistically significant correlations between IL-6 and selected anthropometric and body composition parameters in women from the study and control groups.

Next, correlations between selected anthropometric/body composition parameters and the concentrations of cytokines/adipokines in the saliva of men from the study and control groups (*n* = 39) were analysed. The results are presented in Table 4 and Supplementary Tables S3 and S4.

Statistically significant positive correlations were found between BMI and the concentrations of MMP-9 and resistin in saliva. Statistically significant positive correlations were also shown for waist circumference and the concentrations of MMP-9, IL-6 and resistin in saliva. Furthermore, statistically significant positive correlations were demonstrated between hip circumference and the salivary concentrations of MMP-9 and IL-6. However, a statistically significant positive correlation was demonstrated between the WHR index and the concentrations of MMP-2, IL-6 and resistin. Statistically significant positive correlations were found between subcutaneous fat content (cm^2^) and the salivary concentrations of MMP-9, IL-6 and resistin. However, no statistically significant correlations were found between total body fat content in percentages and kilograms, the content of visceral (cm^2^ and %) and subcutaneous (%) fat, as well as the VAT/SAT ratio and the salivary concentrations of selected cytokines/adipokines.

## 3. Discussion

Chronic low-grade inflammation is one of the mediators of metabolic disturbances associated with obesity. Recent reports have investigated the mechanisms and assessed the impact of proinflammatory cytokines/adipokines on the development of metabolic derangements in obesity [14]. Particular attention has been paid to saliva testing, mainly because of the non-invasive method of its collection and the diagnostic possibilities it offers in routine medical practice. 

MMPs play an important role in the pathological remodelling of blood vessel walls, including vascular endothelial dysfunction, smooth muscle hyperplasia and the formation of unstable atherosclerotic plaques [12]. The present study demonstrated higher concentrations of MMP-2 in the saliva of subjects with obesity compared to individuals with normal body weight. Furthermore, statistically significant differences in the concentrations of MMP-2 in the saliva of obese women compared to women with normal body weight were found (*p* = 0.0012). Similar results were obtained in a study by Mota et al. from 2019, in which salivary concentrations of MMP-2 were statistically significantly (*p* < 0.05) higher in obese subjects compared to individuals with normal body weight [15]. The present study as well as one of our previous investigations [16] revealed similar, positive correlations regarding MMP-2 concentrations in the saliva of obese individuals. We previously demonstrated strong, positive correlations between salivary MMP-2 concentrations and BMI (r = 0.806, *p* < 0.001), total body fat content (TBFkg r = 0.804, *p* < 0.001, TBF% r = 0.794, *p* < 0.001), waist circumference (r = 0.796, *p* < 0.001), visceral fat (VAT cm^2^, r = 0.646, *p* < 0.005) and the VAT/SAT ratio (r = 0.701, *p* < 0.002). The fact that we again obtained statistically significant positive correlations between salivary MMP-2 concentrations, BMI and total body fat content indicates that the concentration of this cytokine may depend on body fat content, but further research exploring the mechanisms of metabolic disturbances that may influence salivary MMP-2 concentrations is needed [16]. 

Our study also showed statistically significantly higher concentrations of MMP-9 in the saliva of subjects with obesity compared to those with normal body weight, both in women (*p* = 0.0451) and men (*p* = 0.0028). Due to a limited number of studies on the concentration of MMP-9 in the saliva of individuals with excessive body weight, we decided to compare the obtained results with blood serum concentrations of MMP-9 reported in the available literature. Andrade et al. revealed statistically significantly (*p* < 0.001) higher concentrations of MMP-9 in the serum of women with primary obesity (*n* = 36) in comparison to women with normal body weight (*n* = 30) [17]. Similar findings were reported by Kosmal et al., who demonstrated a statistically significantly (*p* < 0.001) higher concentration of MMP-9 in the serum of obese women compared to women with normal weight [18]. To the best of our knowledge, the present study is the first to examine MMP-9 concentrations in the saliva of obese individuals, and therefore further studies evaluating the concentration of this cytokine in saliva are recommended. 

Interleukin 6 (IL-6) is a proinflammatory cytokine that modulates the body’s immune response, affecting the function of the nervous, hematopoietic and endocrine systems. IL-6 plays an important role in the pathogenesis of coronary artery disease as well as type 1 and type 2 diabetes. Moreover, IL-6 may increase the risk of cancer in people with excessive body weight [19]. Our study showed higher concentrations of IL-6 in the saliva of obese subjects compared to individuals with normal body weight. Moreover, statistically significant differences were found in the salivary concentrations of this cytokine between men from the study group and men from the control group (*p* = 0.0430). To date, a limited number of reports on the concentration of IL-6 in the saliva of obese people have been published. Furthermore, the reports mainly examined children. A study by Pîrsean et al. demonstrated that the concentrations of IL-6 in the saliva of overweight/obese children were significantly higher than the concentrations of this cytokine in the saliva of children with normal body weight [20]. The study, in contrast to our study, revealed a positive correlation between the salivary IL-6 concentration and BMI. However, in a study by Selvaraju et al., the concentrations of IL-6 in the saliva of obese children were 3.4 times higher compared to children with normal body weight [21]. The above results were confirmed by Roytblat et al. who showed statistically significantly (*p* < 0.05) higher concentrations of IL-6 in the serum of patients with excessive body weight compared to individuals with normal weight [22]. Due to a limited number of studies in adults and inconsistent data regarding children, it is suggested that further research on salivary IL-6 concentrations is conducted in order to elucidate the potential impact of IL-6 levels on metabolic derangements in obesity.

Resistin is an adipokine that may contribute to the development of insulin resistance, type 2 diabetes, hypertension, dyslipidemia and atherosclerosis [23]. Our study demonstrated higher resistin concentrations in the saliva of obese subjects compared to people with normal body weight and a statistically significant difference was found for men from both groups (*p* = 0.0397). Similar results were obtained by Lehmann-Kalata et al., who revealed statistically significantly (*p* = 0.013) higher concentrations of resistin in the saliva of obese patients compared to individuals with normal body weight [24]. Similar results were reported by Al.-Ravi et al., who found statistically significantly higher concentrations of resistin in obese subjects compared to individuals with normal body weight [25]. The difference between the above study and our present investigation is that Al.-Ravi et al. compared obese individuals with patients with diabetes. The latter showed the highest salivary concentrations of resistin [25]. The authors of the study concluded that high levels of resistin in saliva, which are associated with obesity, may be one of the factors predisposing obese individuals to type 2 diabetes. Therefore, it is suggested that further studies exploring the potential impact of high concentrations of resistin on the development of type 2 diabetes are conducted. 

The salivary concentrations of IL-1β were also assessed in the present study. The cytokine, produced by macrophages, impairs insulin signalling and increases lipolysis [26]. To the best of our knowledge, our study is the first to assess the concentrations of this cytokine in the saliva of obese patients. We demonstrated higher concentrations of IL-1β in the saliva of obese patients in comparison to individuals with normal body weight. Furthermore, statistically significantly (*p* = 0.0035) higher concentrations of IL-1β were demonstrated in the saliva of obese men in comparison to men with normal body weight. Similar results were reported by Tvarijonaviciute et al., who revealed that IL-1β concentrations in the saliva of children with excessive body weight were 2.6 times higher compared to children with normal body weight [27]. Therefore, further studies are needed to confirm the benefits of evaluating salivary IL-1β concentrations in obese adults as risk factors for metabolic disturbances associated with obesity. 

The limitation of our study is a small sample size. The main reason for this limitation is the high cost of reagents for the analysis of selected proinflammatory cytokines/adipokines. Furthermore, the authors of the study focused on uncomplicated obesity, which limited the number of people eligible for the study. On the other hand, careful selection of the small sample allowed us to produce accurate results that were not cofounded by other factors. Another limitation of the study is the use of electrical bioimpedance only. The gold standard for assessing body composition is the DEXA (dual-energy X-ray absorptiometry) test. We believe that further studies on larger populations may enable researchers to learn the mechanisms of the development of metabolic disorders related to the concentration of proinflammatory cytokines in the saliva of obese patients and translate the findings into everyday clinical practice.

## 4. Materials and Methods

This study followed an observational design. It included 116 individuals (77 women and 39 men). The study protocol was approved by the Bioethics Committee of the Medical University of Bialystok, Poland (No. R-I-002/647/2019 and APK.002.39.2021). All participants gave written informed consent to participate in the study prior to its commencement. 

Men and women aged 20–55 years with primary obesity were enrolled in the study. An additional inclusion criterion was the absence of periodontal disease and inflammation in the oral cavity. The exclusion criteria were secondary obesity, type 1 and type 2 diabetes, acute coronary artery disease, endocrine disorders, appetite disorders, pregnancy and lactation, use of hormonal contraception or hormone replacement therapy, steroid therapy, antiretroviral therapy, previous surgical or pharmacological treatment for obesity, chronic inflammatory diseases, malignancies, a pacemaker. Women were also asked about the phase of the menstrual cycle and the regularity of menstruation. The analyses in women were performed in the follicular phase (i.e., between the 8th and 11th day of the cycle).

Basic anthropometric measurements (body weight and height) of the study participants were taken, their BMIs were calculated and bioelectrical impedance analysis (BIA) was performed. Based on the obtained results, the participants were divided into two groups: the study group and the control group. The study group consisted of 75 people (46 women and 29 men) with obesity (BMI = 30.0–39.9 kg/m^2^; >30% total body fat content for women and >25% for men). The control group comprised 41 people (31 women and 10 men) with normal body weight (BMI = 18.5–24.9 kg/m^2^, 20–30% total body fat content for women and 15–20% for men). Saliva samples were collected from all study participants to determine the concentrations of selected proinflammatory cytokines and adipokines.

### 4.1. Body Composition Analysis

In order to measure body composition, BIA was performed on all participants using the BioScan 920-2 Analyzer (Essex, UK). It enabled the assessment of the following parameters: total body fat percentage (%), total fat-free mass percentage (%), total body fat mass (kg), total skeletal muscle mass (kg), total body water (L), extracellular (L) and intracellular water (L). The area of adipose tissue in the transverse section of the abdomen was also determined: VAT (in cm^2^ and %) and SAT (in cm^2^ and %), and the VAT/SAT ratio was also determined. Body composition analysis was performed in the morning, following an overnight fast, with no strenuous physical activity prior to the test.

### 4.2. Saliva Sample Collection

Saliva was collected using a standard method. Samples were collected between 9:00 and 11:00 a.m. All subjects abstained from eating and drinking for 2 h prior to sample collection. The subjects rinsed their mouths with deionised water and were sitting in a comfortable position with their eyes open and head titled slightly forward. Unstimulated whole saliva was collected for 10 min by spitting, as described by Navazesh [28]. Saliva samples were homogenised and clarified by centrifugation at 1200 RPMI for 15 min at 4 °C. The aliquots of clarified supernatants were stored at −70 °C for ELISA measurements.

### 4.3. Determination of IL-6, IL-1β, Resistin, MMP-9 and MMP-2

Highly sensitive assay kits (R&D Systems) were used to determine protein concentrations in saliva samples. The tests were performed according to the manufacturer’s recommended protocols. The microtiter plate provided in the kits was pre-coated with a monoclonal antibody specific to the analysed protein. Standards and samples were added to the appropriate microtiter plate wells. Following incubation at room temperature, an enzyme-linked polyclonal antibody was added. Then, the microplate wells were aspirated and washed four times. Next, a substrate solution was added to each well. The enzyme–substrate reaction was terminated by addition of a stop solution and the colour change was measured spectrophotometrically at 450 ± 2 nm. The antigen concentration in the samples was determined by comparing the O.D. to the standard curve. 

### 4.4. Statistical Analysis

Statistical analysis of the obtained results was performed using STATISTICA 13.3 software by StatSoft (version 13.3, Warsaw, Poland). Descriptive statistics were prepared by determining the value of the median and the upper and lower quartiles for quantitative features. A significance level of *p* < 0.05 was assumed. Non-parametric methods were used due to a lack of normal distribution. Two independent samples were compared using the Mann–Whitney *U* test (analysis of the concentrations of adipokines and cytokines in saliva for differences between the study and control groups). Spearman’s rank correlations were used to assess the relationships between the concentrations of proinflammatory parameters and body composition parameters.

## 5. Conclusions

1. Higher concentrations of selected proinflammatory cytokines and adipokines are found in the saliva of obese individuals in comparison to individuals with normal body weight.
2. It is likely that higher concentrations of MMP-2, MMP-9 and IL-1β can be detected in the saliva of obese women compared to non-obese women, while higher concentrations of MMP-9, IL-6 and resistin can be found in the saliva of obese men compared to non-obese men, which suggests that further research to confirm our observations and determine the mechanisms of development of metabolic complications associated with obesity depending on gender is needed.

## Figures and Tables

**Table 1:** Comparison of anthropometric and body composition parameters of women and men from the study and control groups.

Parameter	Women (*n* = 77)				*p* *	Men (*n* = 39)				*p* **
	Study Group (*n* = 46)		Control Group (*n* = 31)			Study Group (*n* = 29)		Control Group (*n* = 10)		
	Median	Q1–Q3	Median	Q1–Q3		Median	Q1–Q3	Median	Q1–Q3	
Age (years)	46.00	39.00–52.00	36.00	30.00–45.00	<0.001 *	44.00	37.00–50.00	37.00	29.00–40.00	0.039 **
Height (cm)	164.25	159.00–169.00	163.00	160.00–170.00	0.897	181.00	175.00–186.00	181.50	174.00–184.00	0.646
Weight (kg)	93.50	88.00–100.40	63.00	58.00–66.00	<0.001 *	110.50	101.00–118.50	80.50	76.00–83.00	<0.001 **
BMI (kg/m^2^)	32.85	32.60–36.60	23.00	21.90–24.50	<0.001 *	33.70	31.80–35.70	24.73	24.30–24.92	<0.001 **
Waist circumference (cm)	107.00	104.00–112.00	81.00	78.00–85.00	<0.001 *	113.00	109.00–117.00	87.00	85.00–94.00	<0.001 **
Hip circumference (cm)	120.00	116.00–124.00	97.00	94.00–101.00	<0.001 *	116.00	113.50–118.00	103.00	101.00–107.00	<0.001 **
WHR	0.92	0.88–0.95	0.84	0.79–0.88	<0.001 *	0.97	0.96–0.99	0.86	0.83–0.88	<0.001 **
Body fat (kg)	42.31	36.99–45.73	16.97	13.85–20.41	<0.001 *	36.77	32.12–41.14	15.46	13.62–18.02	<0.001 **
Body fat (%)	45.42	41.09–47.38	28.37	24.45–30.39	<0.001 *	33.43	30.49–36.14	19.38	16.91–21.24	<0.001 **
VAT (cm^2^)	261.00	179.00–350.00	93.00	65.00–124.00	<0.001 *	296.00	238.50–350.00	120.00	83.00–214.00	<0.001 **
SAT (cm^2^)	127.00	105.00–145.00	72.00	54.00–93.00	<0.001 *	128.50	109.50–149.00	69.50	63.00–74.00	<0.001 **
VAT (%)	68.22	60.36–74.12	56.02	48.33–62.32	<0.001 *	69.43	67.49–74.86	60.71	58.15–76.77	0.1514
SAT (%)	31.77	25.88–39.64	43.98	37.68–51.67	<0.001 *	30.57	25.13–32.51	39.29	23.23–41.85	0.1514
VAT/SAT ratio	2.14	1.52–2.86	1.27	0.94–1.65	<0.001 *	2.27	2.07–2.98	1.54	1.39–3.31	0.1514

Q1–Q3: 1st–3rd quartile, BMI—body mass index, WHR—waist-to-hip ratio, VAT—visceral adipose tissue, SAT—subcutaneous adipose tissue. Statistical significance (*p* < 0.05), *p* *—statistical differences between women from study and control groups, *p* **—statistical differences between men from study and control groups.

**Table 2:** Comparison of the concentration of selected proinflammatory cytokines and adipokines between women and men from the study and control groups.

Parameter	Women (*n* = 77)				*p* *	Men (*n* = 39)				*p* **
	Study Group (*n* = 46)		Control Group (*n* = 31)			Study Group (*n* = 29)		Control Group (*n* = 10)		
	Median	Q1–Q3	Median	Q1–Q3		Median	Q1–Q3	Median	Q1–Q3	
MMP-2 (ng/mL)	0.97	0.75–1.35	0.64	0.50–1.00	0.0012 *	1.16	0.72–1.78	0.74	0.55–1.26	0.1744
MMP-9 (ng/mL)	439.90	274.80–760.50	301.20	171.90–498.40	0.0451 *	519.10	295.20–995.20	178.15	84.60–333.20	0.0028 **
IL-6 (pg/mL)	9.88	4.04–15.23	8.25	3.13–31.70	0.9547	12.88	6.53–22.23	5.85	3.13–9.33	0.0430 **
Resistin (ng/mL)	3.58	1.41–5.03	1.97	0.59–4.56	0.0875	4.76	2.03–6.71	0.94	0.48–4.85	0.0397 **
IL-1β (pg/mL)	390.68	272.43–869.06	239.02	135.35–538.50	0.0035 *	494.70	262.39–1443.07	313.57	109.65–1631.30	0.3484

MMP-2—metalloproteinase-2, MMP-9—metalloproteinase-9, IL-6—interleukin-6, IL-1β—interleukin 1β. Statistical significance (*p* < 0.05), *p* *—statistical differences between women from study and control group, *p* **—statistical differences between men from study and control group.

**Table 3:** Correlations between selected anthropometric/body composition parameters and the concentrations of cytokines/adipokines in the saliva of women from the study and control groups.

Parameter	Women (*n* = 77)				
	MMP-2 (ng/mL)	MMP-9 (ng/mL)	IL-6 (pg/mL)	Resistin (ng/mL)	IL-1β (pg/mL)
BMI (kg/m^2^)	r = 0.460	r = 0.343	r = 0.144	r = 0.258	r = 0.396
	*p* = 0.000 *	*p* = 0.002 *	*p* = 0.208	*p* = 0.023 *	*p* = 0.000 *
Waist circumference (cm)	r = 0.330	r = 0.264	r = 0.069	r = 0.200	r = 0.284
	*p* = 0.003 *	*p* = 0.019 *	*p* = 0.549	*p* = 0.080	*p* = 0.012 *
Hip circumference (cm)	r = 0.355	r = 0.330	r = 0.120	r = 0.247	r = 0.309
	*p* = 0.001 *	*p* = 0.003 *	*p* = 0.296	*p* = 0.029 *	*p* = 0.006 *
WHR	r = 0.171	r = 0.106	r = −0.005	r = 0.049	r = 0.148
	*p* = 0.136	*p* = 0.356	*p* = 0.963	*p* = 0.667	*p* = 0.198
Body fat (kg)	r = 0.387	r = 0.359	r = 0.126	r = 0.224	r = 0.347
	*p* = 0.000 *	*p* = 0.001 *	*p* = 0.273	*p* = 0.049 *	*p* = 0.001 *
Body fat (%)	r = 0.421	r = 0.345	r = 0.136	r = 0.200	r = 0.396
	*p* = 0.000 *	*p* = 0.002 *	*p* = 0.234	*p* = 0.079	*p* = 0.000 *
VAT (cm^2^)	r = 0.373	r= 0.209	r = 0.066	r = 0.305	r = 0.327
	*p* = 0.000 *	*p* = 0.066	*p* = 0.563	*p* = 0.006 *	*p* = 0.003 *
SAT (cm^2^)	r = 0.258	r = 0.287	r = 0.165	r = 0.182	r = 0.275
	*p* = 0.023 *	*p* = 0.011 *	*p* = 0.150	*p* = 0.112	*p* = 0.015 *
VAT (%)	r = 0.305	r = 0.073	r = −0.021	r = 0.216	r = 0.214
	*p* = 0.006 *	*p* = 0.522	*p* = 0.849	*p* = 0.058	*p* = 0.061
SAT (%)	r = −0.073	r = −0.305	r = 0.021	r = −0.216	r = −0.214
	*p* = 0.522	*p* = 0.006 *	*p* = 0.849	*p* = 0.058	*p* = 0.061
VAT/SAT ratio	r = 0.304	r = 0.077	r = −0.019	r = 0.217	r = 0.218
	*p* = 0.007 *	*p* = 0.502	*p* = 0.864	*p* = 0.057	*p* = 0.056

BMI—body mass index, WHR—waist hip ratio, VAT—visceral adipose tissue, SAT—subcutaneous adipose tissue. MMP-2—metalloproteinase-2, MMP-9—metalloproteinase-9, IL-6—interleukin-6, IL-1β—interleukin 1β. *p* *—statistical significance (*p* < 0.05).

**Table 4:** Correlations between selected anthropometric/body composition parameters and the concentrations of cytokines/adipokines in the saliva of men from the study and control groups.

Parameter	Men (*n* = 39)				
	MMP-2 (ng/mL)	MMP-9 (ng/mL)	IL-6 (pg/mL)	Resistin (ng/mL)	IL-1β (pg/mL)
BMI (kg/m^2^)	r = 0.289	r = 0.416	r = 0.282	r = 0.318	r = 0.309
	*p* = 0.073	*p* = 0.008 *	*p* = 0.081	*p* = 0.047 *	*p* = 0.055
Waist circumference (cm)	r = 0.303	r = 0.362	r = 0.360	r = 0.325	r = 0.251
	*p* = 0.060	*p* = 0.023 *	*p* = 0.024 *	*p* = 0.043 *	*p* = 0.122
Hip circumference (cm)	r = 0.179	r = 0.353	r = 0.329	r = 0.310	r = 0.217
	*p* = 0.275	*p* = 0.027 *	*p* = 0.040 *	*p* = 0.054	*p* = 0.183
WHR	r = 0.362	r = 0.297	r = 0.360	r = 0.321	r = 0.239
	*p* = 0.023 *	*p* = 0.066	*p* = 0.024 *	*p* = 0.046 *	*p* = 0.141
Body fat (kg)	r = 0.118	r = 0.300	r = 0.143	r = 0.226	r = 0.104
	*p* = 0.477	*p* = 0.066	*p* = 0.390	*p* = 0.170	*p* = 0.533
Body fat (%)	r = 0.168	r = 0.314	r = 0.120	r = 0.176	r = 0.124
	*p* = 0.312	*p* = 0.054	*p* = 0.470	*p* = 0.290	*p* = 0.456
VAT (cm^2^)	r = 0.147	r = −0.027	r = 0.106	r = −0.174	r = −0.144
	*p* = 0.378	*p* = 0.869	*p* = 0.525	*p* = 0.293	*p* = 0.388
SAT (cm^2^)	r = 0.204	r = 0.353	r = 0.437	r = 0.326	r = 0.263
	*p* = 0.218	*p* = 0.029 *	*p* = 0.005 *	*p* = 0.045 *	*p* = 0.109
VAT (%)	r = 0.138	r = 0.063	r = 0.261	r = −0.002	r = −0.007
	*p* = 0.407	*p* = 0.702	*p* = 0.113	*p* = 0.987	*p* = 0.963
SAT (%)	r = −0.147	r = 0.027	r = −0.106	r = 0.174	r = 0.144
	*p* = 0.378	*p* = 0.869	*p* = 0.525	*p* = 0.293	*p* = 0.388
VAT/SAT ratio	r = 0.151	r = −0.024	r = 0.110	r = −0.178	r = −0.130
	*p* = 0.362	*p* = 0.882	*p* = 0.507	*p* = 0.284	*p* = 0.434

BMI—body mass index, WHR—waist–hip ratio, VAT—visceral adipose tissue, SAT—subcutaneous adipose tissue. MMP-2—metalloproteinase-2, MMP-9—metalloproteinase-9, IL-6—interleukin-6, IL-1β—interleukin 1β. *p* *—statistical significance (*p* < 0.05).

## Data Availability

Data available on request due to restrictions, e.g., privacy, ethical.

## Supplementary Materials

The following supporting information can be downloaded at: https://www.mdpi.com/article/10.3390/ijms24044145/s1.
