# Supplementary material for: Analysis of Selected Salivary Adipokines and Cytokines in Patients with Obesity—A Pilot Study
Source: Int J Mol Sci. 2023 Feb 18;24(4):4145. doi: 10.3390/ijms24044145 (PMC9964799; doi:10.3390/ijms24044145)
Supplement: Supplementary file 1 [file ijms-24-04145-s001.zip › ijms-2185540-supplementary.pdf]

Supplementary Files

Table S1. Correlations between selected anthropometric/body composition parameters and cytokines/adipokines in the saliva of women from the study group.

Women from study group (n=46)					
**Parameter**	**MMP-2 (ng/ml)**	**MMP-9 (ng/ml)**	**IL-6 (pg/ml)**	**Resistin (ng/ml)**	**IL-1β (pg/ml)**
**BMI (kg/m²)**	r=0.321	r=0.329	r=0.341	r=0.189	r=0.289
	p=0.029*	p=0.025*	p=0.020*	p=0.207	p=0.050
**Waist circumference (cm)**	r=0.029	r=0.105	r=0.120	r=0.090	r= - 0.021
	p=0.847	p=0.485	p=0.424	p=0.550	p=0.889
**Hip circumference (cm)**	r=0.095	r=0.288	r=0.201	r=0.121	r=0.065
	p=0.526	p=0.051	p=0.179	p=0.422	p=0.667
**WHR**	r= - 0.042	r= - 0.096	r= - 0.030	r= - 0.050	r= - 0.125
	p=0.781	p=0.525	p=0.841	p=0.741	p=0.405
**Body fat (kg)**	r=0.156	r=0.281	r=0.196	r=0.056	r=0.167
	p=0.299	p=0.058	p=0.191	p=0.711	p=0.264
**Body fat (%)**	r=0.203	r=0.196	r=0.193	r=0.009	r=0.253
	p=0.175	p=0.191	p=0.196	p=0.952	p=0.089
**VAT (cm²)**	r=0.042	r= - 0.037	r=0.034	r=0.215	r=0.115
	p=0.780	p=0.806	p=0.821	p=0.150	p=0.443
**SAT (cm²)**	r=0.079	r=0.283	r=0.208	r= - 0.020	r=0.047
	p=0.600	p=0.056	p=0.163	p=0.890	p=0.752
**VAT (%)**	r=0.057	r= - 0.191	r= - 0.037	r=0.143	r=0.125
	p=0.706	p=0.202	p=0.804	p=0.341	p=0.405
**SAT (%)**	r= -0.057	r=0.191	r=0.037	r= - 0.143	r= - 0.125
	p=0.706	p=0.202	p=0.804	p=0.341	p=0.405
**VAT/SAT ratio**	r=0.061	r= - 0.180	r= - 0.035	r=0.148	r=0.136
	p=0.683	p=0.230	p=0.816	p=0.325	p=0.366

Table S2. Correlations between selected anthropometric/body composition parameters and cytokines/adipokines in the saliva of women from the control group.

Women from control group (n=31)					
**Parameter**	**MMP-2 (ng/ml)**	**MMP-9 (ng/ml)**	**IL-6 (pg/ml)**	**Resistin (ng/ml)**	**IL-1β (pg/ml)**
**BMI (kg/m²)**	r=0.333	r=0.274	r=0.108	r=0.160	r=0.209
	p=0.066	p=0.134	p=0.561	p=0.388	p=0.257
**Waist circumference (cm)**	r=0.032	r=0.233	r=0.086	r= - 0.019	r=0.099
	p=0.860	p=0.206	p=0.642	p=0.916	p=0.596
**Hip circumference (cm)**	r=0.171	r=0.264	r=0.225	r=0.206	r=0.112
	p=0.355	p=0.150	p=0.223	p=0.265	p=0.548
**WHR**	r= - 0.064	r=0.106	r=0.013	r= - 0.067	r=0.146
	p=0.730	p=0.567	p=0.940	p=0.718	p=0.431
**Body fat (kg)**	r=0.176	r=0.484	r=0.270	r=0.217	r=0.119
	p=0.342	p=0.005*	p=0.141	p=0.240	p=0.523
**Body fat (%)**	r=0.305	r=0.552	r=0.282	r=0.236	r=0.227
	p=0.094	p=0.001*	p=0.123	p=0.199	p=0.218
**VAT (cm²)**	r=0.133	r=0.152	r=0.218	r=0.076	r=0.038
	p=0.474	p=0.413	p=0.238	p=0.682	p=0.835
**SAT (cm²)**	r= - 0.031	r=0.155	r=0.358	r=0.178	r=0.265
	p=0.867	p=0.402	p=0.047*	p=0.337	p=0.149
**VAT (%)**	r=0.238	r=0.117	r=0.007	r=0.000	r= - 0.169
	p=0.196	p=0.529	p=0.968	p=0.996	p=0.362
**SAT (%)**	r= -0.238	r= - 0.117	r= - 0.007	r= - 0.000	r=0.169
	p=0.196	p=0.529	p=0.968	p=0.996	p=0.362
**VAT/SAT ratio**	r=0.233	r=0.117	r=0.009	r=0.001	r= - 0.167
	p=0.206	p=0.530	p=0.961	p=0.993	p=0.368

Table S3. Correlations between selected anthropometric/body composition parameters and cytokines/adipokines in the saliva of men from the study group.

Men from study group (n=29)					
**Parameter**	**MMP-2 (ng/ml)**	**MMP-9 (ng/ml)**	**IL-6 (pg/ml)**	**Resistin (ng/ml)**	**IL-1β (pg/ml)**
**BMI (kg/m²)**	r=0.267	r=0.041	r=0.034	r=0.097	r=0.324
	p=0.160	p=0.830	p=0.860	p=0.613	p=0.085
**Waist circumference (cm)**	r=0.206	r=0.012	r=0.095	r=0.079	r=0.190
	p=0.283	p=0.950	p=0.620	p=0.682	p=0.323
**Hip circumference (cm)**	r= - 0.029	r= - 0.010	r=0.077	r=0.051	r=0.110
	p=0.881	p=0.958	p=0.688	p=0.791	p=0.568
**WHR**	r=0.324	r= - 0.052	r=0.114	r=0.171	r=0.219
	p=0.085	p=0.785	p=0.553	p=0.374	p=0.251
**Body fat (kg)**	r= - 0.047	r= - 0.173	r= - 0.265	r= - 0.064	r= - 0.043
	p=0.808	p=377	p=0.171	p=0.744	p=0.827
**Body fat (%)**	r=0.052	r= - 0.158	r= - 0.300	r= - 0.152	r= - 0.002
	p=0.790	p=0.421	p=0.120	p=0.437	p=0.991
**VAT (cm²)**	r= - 0.029	r= - 0.142	r=0.153	r= - 0.040	r=0.026
	p=0.882	p=0.471	p=0.435	p=0.836	p=0.894
**SAT (cm²)**	r=0.025	r= - 0.001	r=0.221	r=0.036	r=0.191
	p=0.898	p=0.994	p=0.256	p=0.855	p=0.330
**VAT (%)**	r=0.044	r= - 0.012	r=0.154	r= - 0.086	r= - 0.103
	p=0.822	p=0.951	p=0.431	p=0.661	p=0.600
**SAT (%)**	r= - 0.044	r=0.012	r= - 0.154	r=0.086	r=0.103
	p=0.822	p=0.951	p=0.431	p=0.661	p=0.600
**VAT/SAT ratio**	r=0.044	r= - 0.020	r=0.152	r= - 0.103	r= - 0.095
	p=0.820	p=0.916	p=0.438	p=0.601	p=0.629

Table S4. Correlations between selected anthropometric/body composition parameters and cytokines/adipokines in the saliva of men from the control group.

Men from control group (n=10)					
**Parameter**	**MMP-2 (ng/ml)**	**MMP-9 (ng/ml)**	**IL-6 (pg/ml)**	**Resistin (ng/ml)**	**IL-1β (pg/ml)**
**BMI (kg/m²)**	r= - 0.291	r=0.285	r=0.332	r=0.595	r=0.182
	p=0.413	p=0.423	p=0.347	p=0.069	p=0.614
**Waist circumference (cm)**	r=0.256	r= - 0.341	r=0.726	r=0.542	r=0.213
	p=0.475	p=0.334	p=0.017*	p=0.105	p=0.553
**Hip circumference (cm)**	r=0.349	r= - 0.460	r=0.436	r=0.300	r=0.190
	p=0.321	p=0.180	p=0.206	p=0.398	p=0.598
**WHR**	r=0.091	r= - 0.422	r=0.596	r=0.055	r= - 0.091
	p=0.800	p=0.224	p=0.068	p=0.879	p=0.800
**Body fat (kg)**	r=- 0.030	r= - 0.018	r=0.625	r=0.393	r=0.151
	p=0.933	p=0.960	p=0.053	p=0.259	p=0.676
**Body fat (%)**	r= - 0.054	r=0.115	r=0.756	r=0.236	r=0.163
	p=0.881	p=0.751	p=0.011*	p=0.510	p=0.651
**VAT (cm²)**	r=0.389	r= - 0.541	r=0.141	r= - 0.291	r= - 0.194
	p=0.266	p=0.106	p=0.697	p=0.413	p=0.590
**SAT (cm²)**	r=0.103	r= - 0.085	r=0.497	r=0.676	r=0.310
	p=0.775	p=0.814	p=0.143	p=0.031*	p=0.381
**VAT (%)**	r=0.151	r= - 0.260	r= - 0.212	r= - 0.600	r= - 0.248
	p=0.676	p=0.467	p=0.555	p=0.066	p=0.488
**SAT (%)**	r= - 0.151	r=0.260	r=0.212	r=0.600	r=0.248
	p=0.676	p=0.467	p=0.555	p=0.066	p=0.488
**VAT/SAT ratio**	r=0.194	r= - 0.273	r= - 0.178	r= - 0.595	r= - 0.231
	p=0.590	p=0.444	p=0.621	p=0.069	p=0.520
